# Long-term care settings in the times of COVID-19: challenges and future directions

**DOI:** 10.1017/S1041610220001416

**Published:** 2020-07-01

**Authors:** Liat Ayalon, Anna Zisberg, Ella Cohn-Schwartz, Jiska Cohen-Mansfield, Silvia Perel-Levin, Elitzur Bar-Asher Siegal

**Affiliations:** 1 Louis and Gabi Weisfeld School of Social Work, Bar Ilan University, Ramat Gan, Israel; 2 Cheryl Spencer Department of Nursing, The University of Haifa, Haifa, Israel; 3 Department of Public Health, Ben Gurion University of the Negev, Beer Sheva, Israel; 4 Department of Health Promotion, School of Public Health, Sackler Faculty of Medicine; The Herczeg Institute on Aging; Igor Orenstein Chair for the Study of Geriatrics; Minerva Center for Interdisciplinary Studies of the End of Life, Tel Aviv University, Tel Aviv, Israel; 5 NGO Committee on Ageing, Geneva; 6 International Network for the Prevention of Elder Abuse (INPEA), Geneva, Switzerland; 7 Language, Logic and Cognition Center, The Hebrew University of Jerusalem, Jerusalem, Israel

The term “long-term care settings” (LTCS) encompasses settings that provide a range of services to meet older persons’ needs for social, personal, and/or health care. These settings may include nursing homes or assisted living facilities, which are designed for people who require assistance in performing activities of daily living, such as bathing or transferring; meals, cleaning services, and social activities are also provided. A skilled nursing facility differs from assisted living in that it aims to meet not only the residents’ physical needs but also their medical needs. Hence, this setting provides in-patient medical treatment and rehabilitation services in addition to the services enumerated above (Sanford *et al.*, 2015). Continuing care retirement communities, on the other hand, represent a residential alternative for older persons, who are independent, when first entering the setting. Residents are free to choose a variety of on-site services, including social activities, health care services, cleaning, and prepared meals. Depending on older persons’ evolving needs, more intensive levels of care might be available to allow older persons to age in place (Ayalon, 2016). Hence, LTCS cater to a varied population of older persons, with very different care needs and resources. The nature of the facilities, the quality of care provided, the cost, and the source of funding may vary dramatically across settings.

## Long-term care institutions as a high-risk environment for older adults during the COVID-19 outbreak

During the COVID-19 outbreak, the infection rate and death rate of long-term care (LTC) residents have been unprecedented (Arons *et al.*, 2020; McMichael, 2020). Recent estimates of the Centre for Disease Control suggest that LTC residents constitute about 27% of all COVID-19 deaths in the United States (https://www.kff.org/medicaid/issue-brief/state-reporting-of-cases-and-deaths-due-to-covid-19-in-long-term-care-facilities/). In Europe, deaths of LTC residents constitute 50% of all COVID-19 deaths (https://www.businessinsider.com/half-europes-covid-19-deaths-in-long-term-care-facilities-2020-4). A recent estimate in Quebec, Canada, suggests that LTC residents constitute as many as 88% of COVID-19 deaths (https://ltccovid.org/wp-content/uploads/2020/06/LTCcovid-country-reports_Canada_June-4-2020.pdf or https://www.thestar.com/politics/federal/2020/05/07/82-of-canadas-covid-19-deaths-have-been-in-long-term-care.html). These statistics likely represent an underestimation, given the fact that not all countries administer post-mortem testing to verify the cause of death. Moreover, sporadic reports about hidden corpses may imply a tendency to under-report COVID-19 deaths in LTCS (https://www.nytimes.com/2020/04/15/nyregion/coronavirus-nj-andover-nursing-home-deaths.html).

Despite the great heterogeneity of the LTC resident population, this population as well as the settings themselves have several features that make LTC residents particularly vulnerable to the current outbreak. Specifically, older age and chronic diseases, which often characterize LTC residents, constitute major risk factors for COVID-19 severe symptoms and death (Jordan *et al.*, 2020). Related to this is the fact that older persons with chronic conditions often present with atypical COVID-19 symptoms (Tay and Harwood, 2020), and thus, in the absence of targeted testing, they are unlikely to receive an adequate diagnosis to prevent further infections.

At the institutional level, it is important to acknowledge the physical characteristics of the settings that were designed to encourage communal living. Older persons, including those who have physical or cognitive impairments, often are expected to share a room with other residents. Moreover, even in settings that house independent older persons, the rooms or residential units are very small with the claim that it encourages contact between the residents in the common areas (Ayalon and Yahav, 2019). These features make physical isolation challenging as residents often cannot self-isolate or if they do self-isolate, they are confined to a very small living area.

Another feature of the setting which puts LTC residents at a greater risk concerns the fact that usually the same care provider provides assistance to multiple care recipients. In the absence of adequate personal protective equipment and practices, the care workers run the risk of transferring the disease across the entire setting and as they usually go back to their homes in the community, they might also carry and transmit the virus across settings. Moreover, many of the institutions are understaffed even during regular times (Stone, 2017). This is because despite being a highly demanding job, care workers receive very limited monetary or social acknowledgment and support (Hussein, 2017). In the light of concerns about the spread of COVID-19 across institutions, staff members were not allowed to work in more than one setting (https://pooranlaw.com/covid-19-government-releases-single-employer-emergency-order-for-long-term-care-sector/), thus, leaving some institutions further understaffed and impairing their ability to follow infection prevention control guidelines. In addition, some institutions had asked their workers to live on-site for a period of up to several weeks to minimize contact with the outside world (https://www.euronews.com/2020/05/04/not-in-my-nursing-home-french-care-home-staff-lock-down-with-residents-to-see-out-covid-1).

## Emotional threats to older adults in long-term care settings during the COVID-19 outbreak

As noted above, LTCS are quite varied in their nature. Whereas some settings provide high-quality services, others might be chronically understaffed and/or provide inadequate care, unrelated to the COVID-19 outbreak. Past research has identified LTC residents as a high-risk group for elder abuse and neglect (Yon *et al.*, 2019). At times of stress, anxiety, and resource constraints, older persons might be even at a greater risk. Moreover, in many countries, the current guidelines on physical isolation do not allow for visits to LTCS. Although the prevention and monitoring of elder abuse and neglect should be well regulated and ongoing within the institution, there is no doubt that family members and outside visitors take an important role in ensuring the quality of services. In the absence of outside visitors, care provided to a particularly vulnerable population in society is not monitored by people outside the setting. This is particularly relevant in the case of elder abuse and neglect, which often occurs behind closed doors.

The emotional and health consequences in this population cannot be underestimated (Armitage and Nellums, 2020). It is expected that in the face of changes in their routine and isolation from family members and loved ones, older LTC residents will experience loneliness, anxiety, distress, and depression. These may not always be alleviated via technological means, especially in those residents with impaired cognitive functioning or low digital literacy. Moreover, if ongoing or new medical and emotional needs of LTC residents are neglected in the current situation as infection prevention and social isolation receive the priority, it may paradoxically increase morbidity and mortality.

Although worries, concerns, anxiety, and loneliness during the COVID-19 outbreak have been reported also in the general population (Costello *et al.*, 2019), it is likely that these negative emotional responses are intensified in LTCS. This is due to several potential reasons. First, LTCS have become notoriously known as high-risk institutions for COVID-19-associated deaths (Pillemer *et al.*, 2020). Hence, it is likely that residents in LTCS would experience higher levels of anxiety as a result. Second, in contrast to older adults in the general population who were “free” to follow or disobey lockdown guidelines as they saw fit, older adults in LTCS were deprived of the opportunity to choose their actions. They were told by the management how to behave during lockdown and were carefully and closely monitored, given the physical characteristics of LTCS. Even settings for independent older adults can easily turn into totalitarian institutions in which the residents have minimal opportunities to exert their will (Ayalon, 2015). Moreover, the small living units of most settings do not resemble a real home as living units are designed to encourage communal living (Green and Ayalon, 2019). Finally, fear of dying in isolation, separated from their loved ones as was often presented in the media may be another source of stress and anxiety (Yardley and Rolph, 2020). These factors might result in higher levels of distress, helplessness, and thus, anxiety and potentially depression among LTC residents.

Physical isolation is particularly challenging for those older persons who are with cognitive decline and dementia and who might not be able to fully comprehend instructions (McCusker *et al.*, 2011; Zimmerman *et al.*, 2014). Agitation, wandering, and physical behaviors can be common accompanying neuropsychiatric symptoms (Lyketsos *et al.*, 2002). This has led to unprecedented rates of chemical and physical restraint use in this population (Graziano Onder *et al.*, 2016), unrelated to the COVID-19 outbreak. Research has unanimously shown the negative effects these restraints have on the health and even mortality of older persons (Stones *et al.*, 2019). During the current outbreak, there is a great risk that restraints will be used as a means to physically isolate those older persons who cannot follow the physical distancing instructions due to their cognitive status.

## Emotional threats to long-term care staff during the COVID-19 outbreak

Health and social care workers have been at the frontline, risking their own lives, during the COVID-19 outbreak (Ng *et al.*, 2020). While the rest of society was under strict lockdown to protect its health, these workers continued to provide services to older adults. The stress potentially experienced by LTC staff may stem from a number of reasons and is somewhat in parallel to older LTC residents. Stories about the heightened vulnerability of LTC residents (Kimball *et al.*, 2020) can be stressful enough. However, because so many of the LTCS were actually affected by the virus, many workers likely had to manage an actual outbreak within the setting, including the death and severe illness of residents. This likely resulted in even higher levels of anxiety, stress, and burnout (Boerner *et al.*, 2017).

Although many LTCS serve high-risk populations, they do not enjoy the same resources as health care settings. Worldwide, there are disturbing stories about LTCS which have been experiencing constant shortages of personal protective equipment (Kruse *et al.*, 2020). In addition, LTC workers had to learn in an expedited way how to use protective gear, as this was not common practice in these settings prior to the COVID-19 outbreak (Gaur *et al.*, 2020; Smith *et al.*, 2008). This likely has put an additional stress on the workers.

In some settings, workers were not allowed to leave for several weeks to protect the residents (https://www.wbur.org/hereandnow/2020/05/18/nursing-home-coronavirus-rvs). Others were asked to work overtime or double-shifts to compensate for the dwindling workforce. Although commendable from the point of view of protecting residents, this is a violation of their working conditions and might result in high levels of burden and burnout. There is plenty of research to show that LTC staff are exposed to precarious working conditions, even in non-emergency situations (Hussein, 2017; Kane, 1994; Österle and Bauer, 2016). Moreover, workers who became sick were not always compensated for their sick time. This likely has put substantial financial hardship on the care staff, many of whom are already financially vulnerable (Hussein, 2017).

On top of these setting-specific concerns, many of the care workers likely have faced stressors related to their family life (Van Houtven *et al.*, 2020). Workers with young children had to manage school closure and identify alternative educational and care environments for their children. Workers who were themselves older or had chronic illness that put them at a higher risk had to either take a leave or stay on the job and risk their lives. Other family situations, such as sick parents or spouse, also required workers to make work-related decisions or sacrifices that the rest of society on lockdown was not forced to make (Van Houtven *et al.*, 2020; Yardley and Rolph, 2020). This likely has exacerbated the sense of anxiety and stress in LTC staff even further.

## What should we do next?

The novel coronavirus is no longer novel. However, in early March when COVID-19 was first defined by the World Health Organization as a pandemic (http://www.euro.who.int/en/health-topics/health-emergencies/coronavirus-covid-19/news/news/2020/3/who-announces-covid-19-outbreak-a-pandemic), this was a new experience to most of us. The past few months have taught all of us important lessons that should guide our future behaviors in the face of a second wave of COVID-19 or any other unknown future virus or other disasters. It is particularly important to acknowledge the good practices and intergenerational solidarity that took place during times of severe trouble (Ayalon *et al.*, 2020). For instance, the use of technology to facilitate communication with family members (Hart *et al.*, 2020) or for video consultations (Greenhalgh *et al.*, 2020) likely will remain and is expected to improve the overall quality of life of LTC residents.

Nevertheless, disconcerting facts and risks related to COVID-19 and LTCS require both research and policy action. Clearly, to allow the rest of society to return to their routine while older persons in LTCS are expected to remain isolated is not only inadequate but is discriminatory and a violation of human rights. At the same time, simply requesting settings to allow family visits without the development of an adequate strategy to prevent the spread of COVID-19 is not a possibility. Efforts to return to a new routine must take into account the physical characteristics of the settings and the views of all stakeholders involved including older persons themselves, their family members, staff, and administrators.

Even without further research, it is clear that additional funding should be directed to support LTCS and ensure the ability of staff to protect themselves and their residents in the face of an impending virus. Although LTCS are considered primarily as non-medical, when it comes to protecting residents from a virus or other pandemic, they should receive the exact same protective gear and training as medical settings. Moreover, ongoing testing is required to facilitate the return of the residents to a routine, which allows them to socially engage and prosper even with the COVID-19 impending risk in the background.

It also is important to identify particularly good and poor case examples to learn about adequate and inadequate strategies to address the COVID-19 outbreak. Currently, there are no consistent public records concerning COVID-19 fatalities in LTC as not all countries record this information, and LTC residency is not consistently accounted for in models that attempt to explain COVID-19 mortality (Pillemer *et al.*, 2020). To better understand the role these settings play during the current COVID-19 outbreak, it is important to record such information and make it accessible to researchers and the public. A case–control study that compares settings with and without COVID-19 cases might assist in identifying the unique features that make some settings more prone to spread COVID-19 than others.

The current outbreak has put LTCS at the center of attention. As the rest of society is gradually adapting to a new routine, it is our duty to ensure that LTCS do not become prisons that prevent older persons from interacting with the rest of society. Human rights should never be a burden to care provision. They are the means to resolving issues, improving service delivery, and improving communication (Care about rights? Section four, information for care providers and care workers, http://careaboutrights.scottishhumanrights.com/section4-page01.html).

The outbreak has resulted in a severe disruption of the residents’ routine. While this might be expected and accepted for a short period of time, this cannot become the new norm. Moreover, we should ensure that next time our society confronts such a virus, older adults will not become trapped in settings that have turned into total institutions under the pretext of an emergency situation. Assistance to LTC residents should now be provided not only to foster physical recovery due to delayed treatment and poor health behaviors during lockdown, but also due to high levels of anxiety, depression, and loneliness likely experienced by the residents. After being intimidated about the potential deadly effects of the virus, returning to normal is likely to be slow and challenging, especially for the most at-risk group in society, namely LTC residents.

While protecting the rights of older persons, it should be remembered that also care providers and carers have human rights that should be respected and protected. The LTC staff likely have experienced high levels of stress, anxiety, and burden in the past few months. Emotional assistance is required to help staff to process the experiences during the pandemic. The past few months have stressed the important role of LTC staff at the frontline of fighting the virus and preserving the lives of older adults. As a society, it is our duty to ensure that the status and benefits associated with direct paid eldercare work are improved so that staff are adequately trained and rewarded for their work.
